# Baloxavir safety and clinical and virologic outcomes in influenza virus-infected pediatric patients by age group: age-based pooled analysis of two pediatric studies conducted in Japan

**DOI:** 10.1186/s12887-023-03841-5

**Published:** 2023-01-21

**Authors:** Nobuo Hirotsu, Hiroki Sakaguchi, Keita Fukao, Satoshi Kojima, Pedro A. Piedra, Kenji Tsuchiya, Takeki Uehara

**Affiliations:** 1 Hirotsu Clinic, Kawasaki, Kanagawa Japan; 2 Biostatistics Center, Shionogi & Co., Ltd, Osaka, Japan; 3 Laboratory for Drug Discovery and Disease Research, Shionogi & Co., Ltd, Osaka, Japan; 4 Medical Affairs Department, Shionogi & Co., Ltd, Osaka, Japan; 5 Department of Molecular Virology and Microbiology and Department of Pediatrics, Baylor College of Medicine, Houston, TX USA; 6 Clinical Research Department, Shionogi & Co., Ltd, Osaka, Japan; 7 Drug Development and Regulatory Science Division, Shionogi & Co., Ltd, 8F, Nissay Yodoyabashi East, 3-3-13 Imabashi, Chuo-ku, Osaka, 541-0042 Japan

**Keywords:** Age group, Baloxavir marboxil, Influenza, Children, Pooled analysis

## Abstract

**Background:**

Anti-influenza treatment is important for children and is recommended in many countries. This study assessed safety, clinical, and virologic outcomes of baloxavir marboxil (baloxavir) treatment in children based on age and influenza virus type/subtype.

**Methods:**

This was a post hoc pooled analysis of two open-label non-controlled studies of a single weight-based oral dose of baloxavir (day 1) in influenza virus-infected Japanese patients aged < 6 years (*n* = 56) and ≥ 6 to < 12 years (*n* = 81). Safety, time to illness alleviation (TTIA), time to resolution of fever (TTRF), recurrence of influenza illness symptoms and fever (after day 4), virus titer, and outcomes by polymerase acidic protein variants at position I38 (PA/I38X) were evaluated.

**Results:**

Adverse events were reported in 39.0 and 39.5% of patients < 6 years and ≥ 6 to < 12 years, respectively. Median (95% confidence interval) TTIA was 43.2 (36.3–68.4) and 45.4 (38.9–61.0) hours, and TTRF was 32.2 (26.8–37.8) and 20.7 (19.2–23.8) hours, for patients < 6 years and ≥ 6 to < 12 years, respectively. Symptom and fever recurrence was more common in patients < 6 years with influenza B (54.5 and 50.0%, respectively) compared with older patients (0 and 25.0%, respectively). Virus titers declined (day 2) for both age groups. Transient virus titer increase and PA/I38X-variants were more common for patients < 6 years.

**Conclusions:**

The safety and effectiveness of single-dose baloxavir were observed in children across all age groups and influenza virus types. Higher rates of fever recurrence and transient virus titer increase were observed in children < 6 years.

**Trial registration:**

Japan Pharmaceutical Information Center Clinical Trials Information JapicCTI-163,417 (registered 02 November 2016) and JapicCTI-173,811 (registered 15 December 2017).

**Supplementary Information:**

The online version contains supplementary material available at 10.1186/s12887-023-03841-5.

## Background

Influenza is a common acute respiratory disease in children that can result in hospitalization and life-threatening complications such as bacterial pneumonia and influenza encephalopathy [1–3]. Vaccination is recommended as the main form of prevention of influenza. In the United States annual vaccination is recommended for children 6 months of age and older [4], and by the World Health Organization in children aged 6 months to 5 years to prevent severe influenza outcomes [5]. Vaccination, however, is not completely effective and is often underutilized [6, 7]. Anti-influenza drug treatments are available for children and should be administered early after disease onset [8, 9]. In Japan, the neuraminidase inhibitors (NAIs) oseltamivir, laninamivir, zanamivir, and peramivir are approved for the treatment of influenza in children [9]. Oseltamivir is the preferred treatment as it is administered orally, however, the clinical benefit of oseltamivir is varied, with reduced benefit observed in children infected with influenza B virus compared with those with influenza A virus of either subtypes [10–12], and in those with oseltamivir-resistant influenza variants [13]. Therefore, additional anti-influenza drugs that are safe and provide clinical benefit to children infected with influenza A and B viruses, and with new mechanisms of action and easily administered formulations, are desired.

Baloxavir marboxil (baloxavir) is a single-dose oral anti-influenza drug that is metabolized to baloxavir acid, the active form, and inhibits the cap-dependent endonuclease activity of the polymerase acidic (PA) protein of influenza A and B viruses [14, 15]. In Japan and the United States, baloxavir is approved for the treatment of influenza in adults and children [16, 17]. Baloxavir is also approved for treating influenza in adults in Europe [18]. In a double-blind randomized controlled study in adults and adolescents with uncomplicated influenza, single-dose baloxavir reduced influenza symptom duration compared with placebo, and reduced virus load more rapidly compared with oseltamivir and placebo [19]. In two open-label studies in Japanese pediatric influenza patients aged < 12 years, a single weight-based dose of baloxavir in tablet formulation [20], or as 2% granules [21], was well tolerated and alleviated influenza symptoms with rapid virus reduction [20, 21] with the pharmacokinetics of baloxavir acid generally within the range of concentrations observed in previous studies of adults and adolescents [20–23]. In a randomized double-blind trial in children aged 1 to < 12 years with influenza, median time to alleviation of symptoms was similar between patients treated with single-dose baloxavir (138.1 hours) compared with oseltamivir twice daily for 5 days (150.0 hours) [24]. Amino acid substitutions of isoleucine at position 38 of the influenza virus PA protein (PA/I38X) associated with reduced susceptibility to baloxavir [14] have been detected in baloxavir-treated patients, mainly with influenza A(H3N2) [19–21, 25]. In clinical studies it can be associated with a transient increase in virus load, although no clear association with a longer time to alleviation of influenza symptoms has been established [19–21, 25]. These primary manuscripts reported clinical and virologic, and safety outcomes of baloxavir in each study, but little information was available for characteristics of those outcomes based on virus type/subtype and each age category.

The objective of this post hoc pooled analysis of influenza virus-infected children from two open-label baloxavir studies [20, 21] was to show for the first time that the safety, clinical, and virologic outcomes of baloxavir treatment by age group (< 6 years; ≥6 to < 12 years) and influenza virus type/subtype. Outcomes by PA/I38X-substituted virus were also assessed.

## Patients and methods

Descriptions of the study designs, patient populations, and safety, clinical, and virologic assessments have been published [20, 21].

### Study design and population

Each study was a multicenter open-label non-controlled study in Japanese pediatric outpatients that occurred during the 2016–17 [20] and 2017–18 influenza seasons [21]. Informed consents were obtained, and the registered studies (JapicCTI-163,417; JapicCTI-173,811) were conducted in accordance with the principles of the Declaration of Helsinki and Good Clinical Practice guidelines.

Patients were enrolled in the baloxavir tablet study if they were ≥ 1 to < 12 years, were capable of swallowing a tablet, and had a body weight ≥ 5 kg and a body mass index < 40 kg/m^2^ [20]. For the baloxavir 2% granule study, patients were < 12 years with a body weight < 20 kg (birth weight ≥ 2500 g if aged < 1 year) [21]. For both studies, patients were eligible if they were diagnosed with influenza illness (confirmed by fever ≥38 °C and by a positive rapid influenza diagnostic test with nasal or throat swabs) and had ≤48 hours between the onset of symptoms (when body temperature first exceeded 37.5 °C) and screening. Patients were excluded at screening if they had severe symptoms of influenza, had risk factors including chronic respiratory disease or a compromised immune system, had received antiviral influenza drugs within 30 days before screening, or had previously received baloxavir.

### Baloxavir treatment

On day 1, patients received a single oral weight-based dose of baloxavir (Shionogi & Co., Ltd., Osaka, Japan) as either a tablet [20] or 2% granules [21] without regard for meals. The investigator or sub-investigator administered baloxavir and performed mouth check of patients immediately after the drug was taken.

### Safety, clinical, and virologic assessments

The incidence and severity of adverse events (AEs), vital signs, and clinical laboratory tests were assessed, and AEs were classified using the Medical Dictionary for Regulatory Activities Version 19.1. Clinical assessments including axillary temperature and severity of two influenza symptoms (cough and nasal discharge/nasal congestion) on a 4-point rating scale (0 = absent to 3 = severe) were recorded in an electronic diary. For virologic assessments, nasopharyngeal swabs (or throat swab if nasopharyngeal swab was not feasible) were collected by investigators (days 1, 2, 3 and/or 4, 6, and 9). Virus type/subtype and virus titer were determined. Co-infection was monitored using swab samples assayed by singleplex quantitative reverse transcription polymerase chain reaction (RT-PCR) for respiratory viruses (including influenza) and bacteria (Additional file 1: Supplementary methods). PA/I38X-substituted virus was detected by next-generation sequencing of total RNA extracted from swab samples [20].

Clinical endpoints included time to illness alleviation (TTIA), time to resolution of fever (TTRF), recurrence of influenza illness symptoms, and recurrence of fever. TTIA was defined as the time from baloxavir administration until the following criteria were met and sustained for ≥21.5 hours: cough and nasal discharge/nasal congestion both assessed as 0 (absent) or 1 (mild) and axillary temperature < 37.5 °C. TTRF was defined as an axillary temperature < 37.5 °C and sustained for ≥12 hours. Recurrence of symptoms was defined as a symptom score of moderate or severe at ≥1 time point after day 4 that was higher than the previous time point. Recurrence of fever was defined as resolution of fever before day 4 but then ≥37.5 °C body temperature after day 4, which was higher than the previous time point. Day 4 was used as the cut-off time based on the timing of transient increases in virus titer.

Virologic endpoints were infectious virus titer (log_10_ 50% tissue culture infective dose [TCID_50_]/ml) at days 1–9, co-infection (defined as a sample positive for influenza virus and for viruses/bacteria other than influenza virus at ≥1 time point), and the presence of PA/I38X-substituted viruses (defined as amino acid changes in PA/I38 occurring between day 1 and the last time point with ≥4 log_10_ virus particles/ml [21]) on paired pre- and last post-treatment swab samples.

### Statistical analysis

Safety was analyzed in the safety population (all patients who received ≥1 dose of the study drug). AEs were summarized by age and system organ class/preferred term. Efficacy was analyzed for the intention-to-treat infected population (all patients who received the study drug who had confirmed diagnosis by RT-PCR of influenza virus infection). Median TTIA and TTRF (with 95% confidence intervals [CI]) were estimated by the Kaplan-Meier method. Patients who did not experience illness alleviation or resolution of fever by the last observation time point were censored at the last observation time point. The recurrence of symptoms and fever was analyzed in the subset of patients with confirmed influenza alleviation or resolution of fever, respectively, before day 4 and is summarized by age and virus type/subtype. Data are presented by age group (< 6 years; ≥6 to < 12 years) and influenza virus type/subtype (A(H1N1)pdm09, A(H3N2), or B); presence or absence of PA/I38X-substituted viruses; and presence or absence of co-infection. Analyses were performed using SAS 9.2 and 9.4 (SAS Institute Inc., Cary, NC, USA).

## Results

### Patient characteristics

A total of 137 influenza virus-infected children were included in the pooled analysis (< 6 years: 56/137 patients [40.9%]; ≥6 to < 12 years: 81/137 patients [59.1%]) (Table 1). Median age was 7 years (range, 0–11), median weight was 20.9 kg (range, 4.0–51.0), the most common influenza virus subtype was A(H3N2) (70.1%), and 42.3 and 85.4% of patients received anti-influenza treatment ≤12 hours and ≤ 24 hours, respectively, from the onset of influenza symptoms. Patients < 6 years had a numerically higher rate of co-infection than those aged ≥6 to < 12 years (44.6% vs 30.9%, respectively; Additional file 2: Table S1). The rate of influenza vaccination was balanced between the two age groups but higher in those < 2 years (30.8%; Additional file 3: Table S2).

**Table 1:** Patient demographics and baseline characteristics by age group (ITTI population)

Variable	< 6 years ***N*** = 56	≥6 to < 12 years ***N*** = 81	Overall ***N*** = 137
Age (years)	2.9 ± 1.7	8.5 ± 1.6	6.2 ± 3.2
Median	3.0	9.0	7.0
Range	0–5	6–11	0–11
Male, *n* (%)	21 (37.5)	43 (53.1)	64 (46.7)
Weight (kg)	13.53 ± 3.80	28.34 ± 7.87	22.29 ± 9.78
Median	14.40	26.60	20.90
Range	4.0–21.3	16.5–51.0	4.0–51.0
Body temperature (°C)	38.84 ± 0.52	38.82 ± 0.63	38.83 ± 0.58
Median	38.80	38.70	38.80
Range	38.0–40.2	38.0–40.5	38.0–40.5
Sum of two symptom scores^a^	2.6 ± 1.3	3.3 ± 1.0	3.0 ± 1.2
Median	3.0	3.0	3.0
Range	0–5	0–6	0–6
Time to treatment from influenza onset, *n* (%)			
≥ 0 to ≤12 hours	23 (41.1)	35 (43.2)	58 (42.3)
> 12 to ≤24 hours	26 (46.4)	33 (40.7)	59 (43.1)
> 24 to ≤36 hours	6 (10.7)	11 (13.6)	17 (12.4)
> 36 to ≤48 hours	1 (1.8)	2 (2.5)	3 (2.2)
Influenza virus type (subtype) based on RT-PCR, *n* (%)			
A(H1N1)pdm09	9 (16.1)	4 (4.9)	13 (9.5)
A(H3N2)	27 (48.2)	69 (85.2)	96 (70.1)
B	16 (28.6)	4 (4.9)	20 (14.6)
A, subtype not specified	2 (3.6)	2 (2.5)	4 (2.9)
Mixed infection	2 (3.6)	2 (2.5)	4 (2.9)
Influenza vaccination^b^, *n* (%)	15 (26.8)	21 (25.9)	36 (26.3)
Co-infection with respiratory virus or bacteria^c^, *n* (%)	25 (44.6)	25 (30.9)	50 (36.5)

Data are presented as mean ± SD unless otherwise stated

*ITTI* intention-to-treat infected, *RT-PCR* reverse transcription polymerase chain reaction, *SD* standard deviation

^a^Cough symptom score and nasal discharge/nasal congestion symptom score

^b^Vaccinated within the last 6 months

^c^Patients positive for influenza virus and positive for viruses or bacteria other than influenza at ≥1 time point. The following viruses were detected: adenovirus, bocavirus, coronavirus HKU1, coronavirus OC43, coronavirus NL63, enterovirus, human metapneumovirus, parainfluenza 1, parainfluenza 2, parainfluenza 4, rhinovirus, RSV-A and RSV-B (details in Additional file 2: Table S1)

### Safety

AEs were reported in 23/59 patients (39.0%) aged < 6 years, and in 32/81 patients (39.5%) aged ≥6 to < 12 years (Table 2). The most common AE was vomiting (all grade 1), occurring in 5/59 patients (8.5%) aged < 6 years, and in 9/81 patients (11.1%) aged ≥6 to < 12 years. All vomiting AEs occurred > 30 minutes after dosing, except for 2 patients aged 6 and 9 years. The frequency of infections and infestations (preferred term by Medical Dictionary for Regulatory Activities Version 19.1) was similar between patients aged ≥2 to < 6 years (7/45 patients; 15.6%) and ≥ 6 to < 12 years (12/81 patients; 14.8%), but was higher in those < 2 years (5/14 patients; 35.7%) (Additional file 4: Table S3). All AEs were of mild or moderate severity (grade 1 or 2) [20, 21].

**Table 2:** Adverse events occurring in ≥2% of patients in any age group (safety population)

System organ class Preferred term	Age group		
	< 6 years ***N*** = 59	≥6 to < 12 years ***N*** = 81	Overall ***N*** = 140
	***n*** (%)	***n*** (%)	***n*** (%)
Patients with any AEs^a^	23 (39.0)	32 (39.5)	55 (39.3)
Infections and infestations	12 (20.3)	12 (14.8)	24 (17.1)
Bronchitis	2 (3.4)	1 (1.2)	3 (2.1)
Nasopharyngitis	3 (5.1)	0	3 (2.1)
Pharyngitis	0	3 (3.7)	3 (2.1)
Otitis media	2 (3.4)	0	2 (1.4)
Sinusitis	0	2 (2.5)	2 (1.4)
Upper respiratory tract infection	2 (3.4)	0	2 (1.4)
Bacterial infection	0	2 (2.5)	2 (1.4)
Oral herpes	0	2 (2.5)	2 (1.4)
Nervous system disorders	0	2 (2.5)	2 (1.4)
Headache	0	2 (2.5)	2 (1.4)
Respiratory, thoracic and mediastinal disorders	4 (6.8)	2 (2.5)	6 (4.3)
Upper respiratory tract inflammation	2 (3.4)	0	2 (1.4)
Gastrointestinal disorders	7 (11.9)	16 (19.8)	23 (16.4)
Vomiting^b^	5 (8.5)	9 (11.1)	14 (10.0)
Constipation	1 (1.7)	2 (2.5)	3 (2.1)
Diarrhea	0	3 (3.7)	3 (2.1)
Skin and subcutaneous tissue disorders	4 (6.8)	1 (1.2)	5 (3.6)
Dry skin	2 (3.4)	0	2 (1.4)
Injury, poisoning and procedural complications	0	2 (2.5)	2 (1.4)
Ligament sprain	0	2 (2.5)	2 (1.4)

Preferred term by Medical Dictionary for Regulatory Activities Version 19.1

*AE* adverse event

^a^Includes all AEs regardless of frequency

^b^All vomiting AEs occurred > 30 minutes after dosing, except for two patients aged 6 and 9 years

### Clinical outcomes

**Fig. 1:**
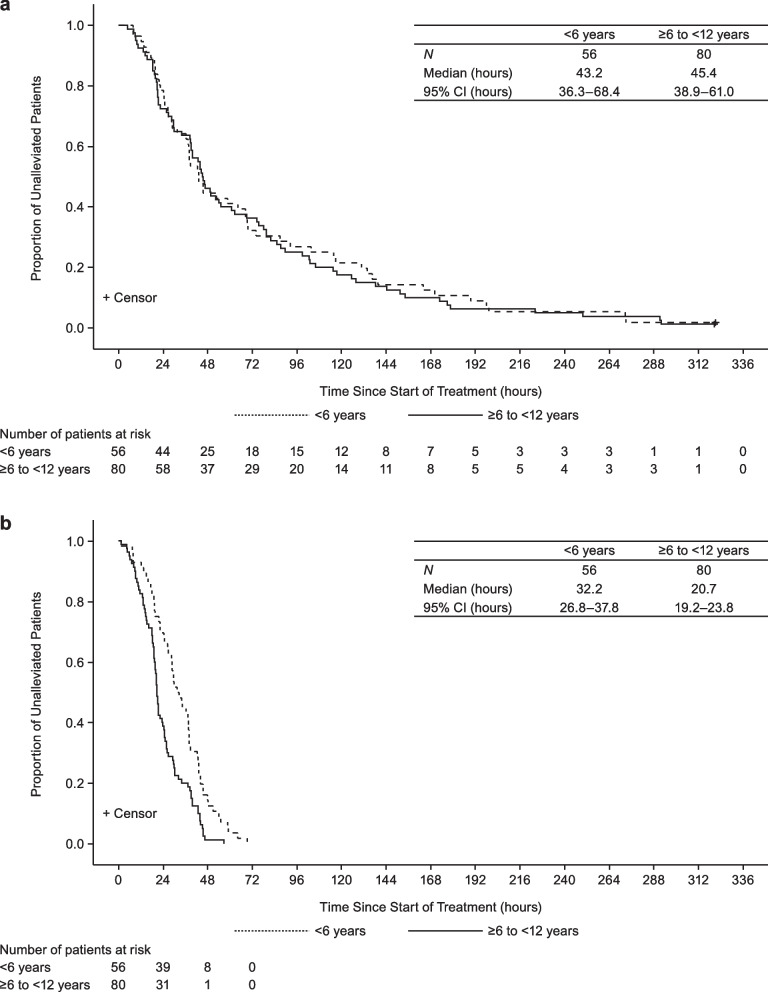
TTIA and TTRF after treatment with baloxavir. Kaplan-Meier analysis of (**a**) time to illness alleviation and (**b**) time to resolution of fever, after treatment with baloxavir. Influenza illness was composed of cough, nasal discharge/nasal congestion, and elevated body temperature. Patients who did not experience alleviation of influenza illness or resolution of fever by the last observation time point were censored at the last observation time point. CI: confidence interval; TTIA: time to illness alleviation; TTRF: time to resolution of fever

Median TTIA was 43.2 (95% CI, 36.3–68.4) hours for patients < 6 years and 45.4 (95% CI, 38.9–61.0) hours for patients ≥6 to < 12 years (Fig. 1a). Median TTRF was 32.2 (95% CI, 26.8–37.8) for patients < 6 years and 20.7 (95% CI, 19.2–23.8) for patients ≥6 to < 12 years (Fig. 1b). Within each age group, median TTIA and TTRF were similar in those infected with influenza A(H3N2) or B (Additional file 5: Table S4).

Recurrence of influenza symptoms after day 4 was more common for patients < 6 years infected with influenza B than for patients ≥6 to < 12 years (Table 3). Fever recurrence after day 4 was more common in patients < 6 years infected with influenza A(H3N2) or B compared with older patients (Table 3).

**Table 3:** Recurrence of influenza symptoms and fever by age group and influenza virus type (subtype)

Influenza virus type (subtype) Variable	< 6 years % (***n/N***)	≥6 to < 12 years % (***n/N***)
A(H1N1)pdm09		
Symptom recurrence after day 4^a^	16.7 (1/6)	0.0 (0/1)
Fever recurrence after day 4^b^	11.1 (1/9)	25.0 (1/4)
A(H3N2)		
Symptom recurrence after day 4^a^	21.1 (4/19)	25.6 (11/43)
Fever recurrence after day 4^b^	29.6 (8/27)	5.9 (4/68)
B		
Symptom recurrence after day 4^a^	54.5 (6/11)	0.0 (0/3)
Fever recurrence after day 4^b^	50.0 (8/16)	25.0 (1/4)

Patients in the ITTI population with a single influenza virus type (subtype) infection were included in the analysis

*ITTI* intention-to-treat infected

^a^The symptom score (cough and nasal discharge/nasal congestion) was assessed as moderate or severe at least once after day 4 and the score increase was observed compared with the previous time point. The subset of patients with confirmed alleviation of influenza illness before day 4 was included in this analysis. Alleviation of symptoms was defined as when the following criteria were met and sustained for ≥21.5 hours: cough and nasal discharge/nasal congestion both assessed as absent or mild, and axillary temperature < 37.5 °C

^b^37.5 °C or more in body temperature was observed after day 4 and body temperature was increased compared with the previous time point. The subset of patients with confirmed resolution of fever before day 4 was included in this analysis. Resolution of fever was defined as an axillary temperature < 37.5 °C and sustained for ≥12 hours

### Virologic outcomes

Mean infectious virus titers declined within 1 day after baloxavir treatment (day 2) for both age groups and all influenza virus types/subtypes (Table 4). After day 3, a temporary increase in mean infectious virus titer was observed for patients < 6 years for all influenza virus types/subtypes, with highest mean post-baseline titer observed in patients with influenza B at day 4 (Table 4). At day 9, mean infectious virus titers for patients ≥6 to < 12 years were below the lower level of detection (0.7 log_10_ TCID_50_/ml) for all virus types/subtypes; for patients < 6 years, mean virus titers remained near the level of detection for those infected with influenza A(H1N1)pdm09 (0.83 log_10_ TCID_50_/ml) or A(H3N2) (1.25 log_10_ TCID_50_/ml) (Table 4).

**Table 4:** Summary of viral titer (log_10_ TCID_50_/ml) by age group and influenza virus type (subtype)

Study day		A(H1N1)pdm09		A(H3N2)		B	
		< 6 years ***N*** = 9	≥6 to < 12 years ***N*** = 4	< 6 years ***N*** = 27	≥6 to < 12 years ***N*** = 69	< 6 years ***N*** = 16	≥6 to < 12 years ***N*** = 4
Day 1	*n*	8	4	27	69	16	4
	Mean (SD)	6.34 (1.70)	5.65 (2.12)	5.29 (1.56)	5.22 (1.99)	6.02 (1.35)	4.35 (2.55)
Day 2	*n*	8	4	27	69	16	4
	Mean (SD)	0.96 (0.54)	1.40 (1.40)	1.04 (0.82)	0.83 (0.44)	2.14 (1.47)	1.65 (1.32)
Day 3	*n*	6	1	18	39	7	3
	Mean (SD)	0.70 (0)	1.3 (−)^a^	0.75 (0.15)	0.75 (0.24)	2.69 (1.63)	2.30 (2.77)
Day 4	*n*	4	3	11	41	10	2
	Mean (SD)	1.10 (0.62)	0.80 (0.17)	1.28 (1.34)	0.78 (0.29)	3.98 (1.96)	0.70 (0)
Day 6	*n*	8	4	27	69	16	4
	Mean (SD)	0.83 (0.35)	1.60 (1.43)	2.07 (1.37)	0.86 (0.62)	2.19 (1.52)	0.70 (0)
Day 9	*n*	8	4	27	69	16	4
	Mean (SD)	0.83 (0.35)	0.70 (0)	1.25 (1.10)	0.70 (0)	0.70 (0)	0.70 (0)

Detection limit of virus titer: 0.7 log_10_ TCID_50_/ml

The subset of patients who were positive for influenza virus titer at baseline was included in this analysis

*SD* standard deviation; TCID_50_: 50% tissue culture infective dose

^a^SD not calculable because *n* = 1

### Amino acid substitutions at PA/I38

**Fig. 2:**
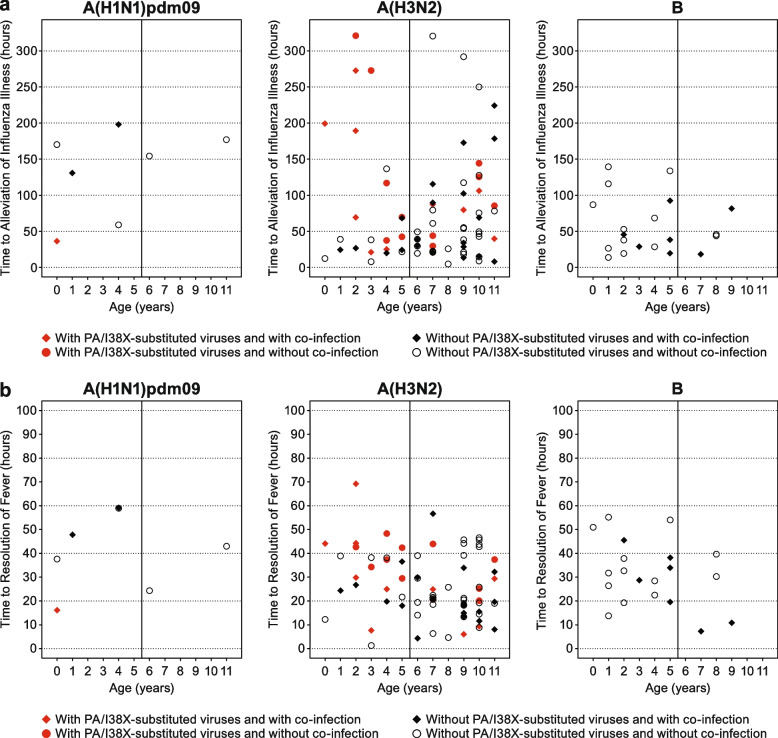
TTIA and TTRF by virus type/subtype, with/without PA/I38X-substituted viruses. Scatterplots of (**a**) time to alleviation of influenza illness and (**b**) time to resolution of fever, after treatment with baloxavir in patients with and without PA/138X-substituted viruses and with or without co-infection, by age and influenza virus type/subtype. For patients with influenza A, the subset of patients with paired sequencing at both baseline and post-treatment was included in this analysis. For patients with influenza B, patients in the ITTI population were included in this analysis. ITTI: intention-to-treat infected; PA/I38X: polymerase acidic protein variants at position I38; TTIA: time to illness alleviation; TTRF: time to resolution of fever

Of the patients with pre- and post-treatment samples, the proportion of patients with PA/I38X-substituted viruses was higher in patients < 6 years than in patients ≥6 to < 12 years with influenza A(H1N1)pdm09 (20.0% vs 0.0%, respectively) and influenza A(H3N2) (52.2% vs 18.9%, respectively). PA/I38X-substituted viruses were not observed in patients infected with influenza B in either age group (Table 5). There was no clear association between virus type/subtype, PA/I38X detection, or co-infection with TTIA or TTRF (Fig. 2). PA/I38X-substituted virus was detected in a small subset of younger patients (< 6 years) with influenza A(H3N2) and a longer TTIA.

**Table 5:** Emergence of PA/I38X-substituted influenza virus

Influenza virus type (subtype) PA/I38X-substituted virus	< 6 years % (***n/N***)	≥6 to < 12 years % (***n/N***)
A(H1N1)pdm09		
ITTI population	11.1 (1/9)	0.0 (0/4)
Patients with paired sequence data	20.0 (1/5)	0.0 (0/2)
A(H3N2)		
ITTI population	44.4 (12/27)	14.5 (10/69)
Patients with paired sequence data	52.2 (12/23)	18.9 (10/53)
B		
ITTI population	0.0 (0/16)	0.0 (0/4)
Patients with paired sequence data	0.0 (0/13)	0.0 (0/3)

*ITTI* intention-to-treat infected, *PA/I38X* polymerase acidic protein variant at position I38

## Discussion

Anti-influenza treatment is important for children, particularly those < 5 years or those with at risk conditions who are at a higher risk of developing complications than older healthy children and adults [26], and for school-aged children who are important contributors to influenza transmission [27]. This post hoc pooled analysis of influenza virus-infected children from two studies of single-dose baloxavir [20, 21] is the first study to show the safety and effectiveness of baloxavir in pediatric patients by age group (< 6 years; ≥6 to < 12 years). The overall frequency of AEs was similar in both age groups; however, the frequency of AEs categorized as infections and infestations was higher in the children < 6 years, which was attributed to the higher incidence in children < 2 years (Additional file 4: Table S3). The higher incidence of AEs in children < 2 years was considered due to the infection itself and the children’s immature immune systems, and not resulting from administration of baloxavir. Of note, no severe AEs were reported in any age group. These results suggest baloxavir was well tolerated in all age groups.

In this study, median TTIA was similar among the age groups (< 6 years: 43.2 hours; ≥6 to < 12 years: 45.4 hours) even though a temporary virus titer increase was observed mainly in younger children. Because our studies did not contain a control group, to estimate baloxavir effectiveness we compared our findings with previous NAI studies in children. Most patients in our studies were infected with influenza A(H3N2), and a comparison of patients aged < 6 years with influenza A(H3N2) with those aged 3–9 years treated with laninamivir or oseltamivir suggests baloxavir’s effectiveness in reducing TTIA (median: 38.9 hours with baloxavir vs 88.6 hours with laninamivir and 44.3 hours with oseltamivir) [28]. Median TTRF for both baloxavir-treated age groups was shorter than that reported for both placebo-treated (median: 2.9 days [approximately 69.6 hours]) and oseltamivir-treated (median: 1.7 days [approximately 40.8 hours]) pediatric patients aged 1–3 years in a randomized trial [29]. However, given several differences in the setting of studies and limited sample size with no statistical comparisons, no firm conclusion can be drawn directly via these comparisons. Of particular note, baloxavir is administered as a single oral dose, including a granule formulation for infants, whereas laninamivir is administered as an inhalant, and oseltamivir requires twice-daily dosing for 5 days; therefore, baloxavir should be a new efficacious treatment option for influenza in children that may enhance adherence by single-dose oral administration [30].

Recurrence of fever is observed in > 25% of children aged < 12 years in the natural course of influenza [31]. Particularly, higher frequencies of biphasic fever in children aged < 9 years infected with influenza B (10–40%) compared with those infected with influenza A have been observed with oseltamivir or laninamivir treatment [32, 33], which may be explained in part by the lower susceptibility of influenza B to oseltamivir in young children [10] or to the immature immune systems of younger children [32]. Likewise, in the present study, recurrence in fever or symptoms was observed in baloxavir-treated patients predominantly in those aged < 6 years infected with influenza A(H3N2) or B. Recurrence of symptoms in baloxavir-treated patients aged ≥6 to < 12 years was observed, but only for those infected with influenza A(H3N2). The absence of symptom recurrence for those infected with influenza A(H1N1)pdm09 or B may be a consequence of the small patient numbers infected with these strains in this study and needs to be investigated further. Although fever and symptom recurrence during or after increases in virus titer was observed in some patients in this study, the titer increases were transient and all patients with symptom recurrence recovered without the need for additional anti-influenza treatment. Of note, the transient virus titer increase did not always reflect symptom changes. This may be explained by the fact that the mean virus titers during transient increases were 2–3 log lower than baseline titers and therefore may not have affected patients’ symptoms.

Treatment-emergent PA/I38X-substituted influenza viruses appear to be more common in baloxavir-treated pediatric patients aged < 12 years, occurring at rates of 19.2–23.4%, compared with adult and adolescent influenza patients (2.2–9.7%) [19–21]. Emergence of viruses resistant to anti-influenza treatment is inevitable due to the error-prone properties of influenza virus replication [34]. Generally, a higher rate of resistant viruses is observed in pediatric patients. In this study, 52.2% of patients with paired sequence data aged < 6 years with influenza A(H3N2) had treatment-emergent PA/I38X-substituted viruses, which was higher than patients aged 6 to < 12 years (10/53 patients, 18.9%; Table 5) and adults and adolescents with influenza A(H3N2) (35/341 patients, 10.3%) [35]. Despite the higher frequency of PA/I38X-substituted virus in younger patients, rapid viral reduction did occur after baloxavir treatment, and symptom alleviation and resolution of fever without prolongation were observed in most patients. Only a small subset of younger patients with influenza A(H3N2) and PA/I38X-substituted virus exhibited longer TTIA. For patients infected with influenza A(H1N1)pdm09 and B, the incidence of PA/I38X-substituted virus in children aged 6 to < 12 years was similar to that of patients ≥12 years for A(H1N1)pdm09 (4/116 patients, 3.4%) and B (0/87 patients, 0.0%) [35]. Similar to baloxavir treatment in younger patients, emergence of oseltamivir-resistant influenza viruses has been shown to occur at a higher frequency in children < 5 years compared with older patients [36]. We have previously hypothesized [20] that the higher PA/I38X substitution rate in baloxavir-treated influenza virus-infected children was associated with low baseline antibody titer for influenza, suggesting that the immature immune system cannot suppress the emergence of PA/I38X-substituted viruses. While influenza virus resistance to antivirals in younger children is common [21, 36], data for baloxavir resistance are still limited; therefore, the careful monitoring of resistance to baloxavir is important from a public health perspective.

The pooling of two study populations enabled us to analyze safety, clinical, and virologic outcomes by age group and influenza virus type/subtype in baloxavir-treated pediatric patients. The study was also enhanced by the range of parameters that were assessed (e.g., TTIA, TTRF, recurrence of symptoms and fever, virus titer, and emergence of PA/I38X-substituted virus), allowing for some association between the clinical and virologic outcomes to be made.

Limitations of this study include the post hoc nature of this analysis, the lack of a control group, and the imbalance in patients with influenza A(H1N1)pdm09 and B across the age groups. This is likely due to the fact that the two studies were conducted in different influenza seasons and the granule study contributed most of the younger patients.

Another limitation is the detection of co-infection. We conducted a reverse transcription PCR against 20 respiratory microorganisms from nasal/throat swab samples to detect co-infection (Additional file 1: Supplementary methods). Since respiratory microorganisms other than tested or microorganisms in other infection sites like a gastrointestinal tract were not detectable by this method, we could not rule out the possibility of fever recurrence or prolongation of influenza symptoms caused by co-infection.

## Conclusions

In this pooled analysis, favorable safety and effectiveness of single-dose baloxavir were observed in influenza virus-infected children across all age groups. Fever recurrence and transient increases in influenza virus titer were observed in children < 6 years. In our study, baloxavir-treated children recovered without prolongation of influenza symptoms regardless of age, transient influenza virus titer increase, PA/I38X virus detection, or co-infection.

## Supplementary Information

**Additional file 1.**

**Additional file 2: Table S1.** Co-infected respiratory virus (ITTI population).

**Additional file 3: Table S2.** Patient demographics and baseline characteristics by age groups < 2 years, ≥2 to < 6 years, and ≥ 6 to < 12 years (ITTI population).

**Additional file 4: Table S3.** Adverse events occurring in ≥2% of patients in any age group (safety population).

**Additional file 5: Table S4.** Duration of symptoms and fever by age group and influenza virus type (subtype) (ITTI population *N* = 137).

**Additional file 6: Table S5.** List of IRBs that gave ethical approval for the clinical trials JapicCTI-163,417 and JapicCTI-173,811.

## Data Availability

The datasets used and/or analysed during the current study are available from the corresponding author on reasonable request. Shionogi & Co., Ltd. is committed to disclosing the results of its clinical trials and sharing the clinical trial data with researchers on reasonable request. For further details, please refer to the websites of Shionogi & Co., Ltd. (https://www.shionogi.com/shionogi/global/en/company/policies/shionogi-group-clinical-trial-data-transparency-policy.html) and Vivli (https://vivli.org/).

## Abbreviations

AE: Adverse event
CI: Confidence interval
NAI: Neuraminidase inhibitor
PA: Polymerase acidic
PA/I38X: Polymerase acidic protein variant at position I38
RT-PCR: Reverse transcription polymerase chain reaction
TCID_50_: 50% tissue culture infective dose
TTIA: Time to illness alleviation
TTRF: Time to resolution of fever
