# Supplementary material for: Baloxavir safety and clinical and virologic outcomes in influenza virus-infected pediatric patients by age group: age-based pooled analysis of two pediatric studies conducted in Japan
Source: BMC Pediatr. 2023 Jan 21;23:35. doi: 10.1186/s12887-023-03841-5 (PMC9860230; doi:10.1186/s12887-023-03841-5)
Supplement: Supplementary file 1 — Additional file 1. [file 12887_2023_3841_MOESM1_ESM.docx]

**Additional file 1: Supplementary methods**

Co-infection, defined as an infection with both influenza viruses and other respiratory viruses at any time point during the first 9 days post-enrolment, was monitored using nasal or throat swab samples assayed by singleplex reverse transcription polymerase chain reaction (RT-PCR) for 20 respiratory microorganisms, including influenza viruses and bacteria.

All nasal/throat swab samples were extracted in the designated pre-amplification space using an automated Qiacube robotic platform. The extracted RNA or DNA was then tested by a singleplex one-step quantitative RT-PCR (one-step qRT-PCR). In one-step qRT-PCR, reverse transcription and PCR amplification occurred in the same tube. Gene-specific primers were used for generating the complementary DNA (cDNA) and for subsequent cDNA amplification. During the reverse transcription phase, the RNA was converted into cDNA by reverse transcriptase enzyme. Taq polymerase then amplified the target sequence from the cDNA to a PCR product. The PCR set-up and addition of RNA were done in predesignated areas. The amplified products were never moved into pre-amplification areas. Forty-five samples were tested in each run of the singleplex RT-PCR test along with positive, negative, and no-template controls. A housekeeping gene, *RNase P*, was used as an internal control to determine the quality of the samples. Samples with cycle threshold values ≥37 were re-extracted and retested. PCR data were analyzed, quality checked, and exported electronically to the laboratory database, StoneBase. Quality checks were also performed after data transfer upload to ensure the data were uploaded correctly.

**List of viruses and bacteria tested for co-infection**

| **No.** | **Virus** |
| --- | --- |
| 1 | Influenza A |
| 2 | Influenza B |
| 3 | Influenza C |
| 4 | Adenovirus |
| 5 | Bordetella pertussis |
| 6 | Coronavirus 229E |
| 7 | Coronavirus HKU1 |
| 8 | Coronavirus NL63 |
| 9 | Coronavirus OC43 |
| 10 | Enterovirus |
| 11 | Human bocavirus type 1 |
| 12 | Human metapneumovirus |
| 13 | Mycoplasma pneumoniae |
| 14 | Parainfluenza 1 |
| 15 | Parainfluenza 2 |
| 16 | Parainfluenza 3 |
| 17 | Parainfluenza 4 |
| 18 | Rhinovirus |
| 19 | Respiratory syncytial virus A |
| 20 | Respiratory syncytial virus B |
