# Supplementary material for: Baloxavir safety and clinical and virologic outcomes in influenza virus-infected pediatric patients by age group: age-based pooled analysis of two pediatric studies conducted in Japan
Source: BMC Pediatr. 2023 Jan 21;23:35. doi: 10.1186/s12887-023-03841-5 (PMC9860230; doi:10.1186/s12887-023-03841-5)
Supplement: Supplementary file 2 — Additional file 2: Table S1. Co-infected respiratory virus (ITTI population). [file 12887_2023_3841_MOESM2_ESM.docx]

**Additional file 2: Table S1** Co-infected respiratory virus (ITTI population)

|  | **<6 years**  ***N* = 56** | **≥6 to <12 years** | **Overall**  ***N* = 137** |
| --- | --- | --- | --- |
|  |  | ***N* = 81** |  |
| Number of patients co-infected with respiratory virus | 25 | 25 | 50 |
| Number of viruses detected | 35 | 29 | 64 |
| Adenovirus | 3 | 1 | 4 |
| Bocavirus | 7 | 2 | 9 |
| Coronavirus | 10^a1^ | 9^a2^ | 19 |
| Enterovirus | 3 | 2 | 5 |
| Human metapneumovirus | 0 | 1 | 1 |
| Parainfluenza | 5^a3^ | 3^a4^ | 8 |
| Rhinovirus | 2 | 9 | 11 |
| RSV | 5^a5^ | 2^a5^ | 7 |
| Rhinovirus/Enterovirus^a^ | 1 | 1 | 2 |

Patients positive for influenza virus and positive for viruses or bacteria other than influenza at ≥1 time point

ITTI: intention-to-treat infected

^a^ Not discriminable

^a1^ Coronavirus HKU1 (*n* = 4); coronavirus OC43 (*n* = 1); coronavirus NL63 (*n* = 5)

^a2^ Coronavirus OC43 (*n* = 1); coronavirus NL63 (*n* = 8)

^a3^ Parainfluenza 1 (*n* = 1); parainfluenza 2 (*n* = 3); parainfluenza 4 (*n* = 1)

^a4^ Parainfluenza 2 (*n* = 3)

^a5^ All RSV-A
