# Supplementary material for: Baloxavir safety and clinical and virologic outcomes in influenza virus-infected pediatric patients by age group: age-based pooled analysis of two pediatric studies conducted in Japan
Source: BMC Pediatr. 2023 Jan 21;23:35. doi: 10.1186/s12887-023-03841-5 (PMC9860230; doi:10.1186/s12887-023-03841-5)
Supplement: Supplementary file 3 — Additional file 3: Table S2. Patient demographics and baseline characteristics by age groups < 2 years, ≥2 to < 6 years, and ≥ 6 to < 12 years (ITTI population). [file 12887_2023_3841_MOESM3_ESM.docx]

**Additional file 3: Table S2** Patient demographics and baseline characteristics by age groups <2 years, ≥2 to <6 years, and ≥6 to <12 years (ITTI population)

| **Variable** | **<2 years**  ***N* = 13** | | **≥2 to <6 years**  ***N* = 43** | | **≥6 to <12 years**  ***N* = 81** |
| --- | --- | --- | --- | --- | --- |
| Age (years) | 0.5 ± 0.5 | | 3.6 ± 1.2 | | 8.5 ± 1.6 |
| Median | 1.0 | | 4.0 | | 9.0 |
| Range | 0–1 | | 2–5 | | 6–11 |
| Male, *n* (%) | 3 (23.1) | | 18 (41.9) | | 43 (53.1) |
| Weight (kg) | 8.41 ± 1.90 | | 15.08 ± 2.70 | | 28.34 ± 7.87 |
| Median | 8.40 | | 15.10 | | 26.60 |
| Range | 4.0–12.7 | | 8.8–21.3 | | 16.5–51.0 |
| Body temperature (°C) | 38.95 ± 0.46 | | 38.81 ± 0.54 | | 38.82 ± 0.63 |
| Median | 39.00 | | 38.70 | | 38.70 |
| Range | 38.2–39.9 | | 38.0–40.2 | | 38.0–40.5 |
| Sum of two symptom scores^a^ | 2.6 ± 1.0 | | 2.6 ± 1.3 | | 3.3 ± 1.0 |
| Median | 3.0 | | 3.0 | | 3.0 |
| Range | 1–4 | | 0–5 | | 0–6 |
| Time to treatment from influenza onset, *n* (%) | |  | | |  |
| ≥0 to ≤12 hours | 5 (38.5) | | 18 (41.9) | | 35 (43.2) |
| >12 to ≤24 hours | 8 (61.5) | | 18 (41.9) | | 33 (40.7) |
| >24 to ≤36 hours | 0 | | 6 (14.0) | | 11 (13.6) |
| >36 to ≤48 hours | 0 | | 1 (2.3) | | 2 (2.5) |
| Influenza virus type(subtype) based on RT-PCR, *n* (%) | | | |  | |
| A(H1N1)pdm09 | 4 (30.8) | | 5 (11.6) | | 4 (4.9) |
| A(H3N2) | 4 (30.8) | | 23 (53.5) | | 69 (85.2) |
| B | 5 (38.5) | | 11 (25.6) | | 4 (4.9) |
| A, subtype not specified | 0 | | 2 (4.7) | | 2 (2.5) |
| Mixed infection | 0 | | 2 (4.7) | | 2 (2.5) |
| Influenza vaccination^b^, *n* (%) | 4 (30.8) | | 11 (25.6) | | 21 (25.9) |
| Co-infection with respiratory virus or bacteria^c^, *n* (%) | 4 (30.8) | | 21 (48.8) | | 25 (30.9) |

Data are presented as mean ± SD unless otherwise stated

ITTI: intention-to-treat infected; RT-PCR: reverse transcription polymerase chain reaction; SD: standard deviation

^a^ Cough symptom score and nasal discharge/nasal congestion symptom score

^b^ Vaccinated within the last 6 months

^c^ Patients positive for influenza virus and positive for viruses or bacteria other than influenza at ≥1 time point. The following viruses were detected: adenovirus, bocavirus, coronavirus HKU1, coronavirus OC43, coronavirus NL63, enterovirus, human metapneumovirus, parainfluenza 1, parainfluenza 2, parainfluenza 4, rhinovirus, and RSV-A (details in Additional file 5: Table S4)
