# Supplementary material for: Baloxavir safety and clinical and virologic outcomes in influenza virus-infected pediatric patients by age group: age-based pooled analysis of two pediatric studies conducted in Japan
Source: BMC Pediatr. 2023 Jan 21;23:35. doi: 10.1186/s12887-023-03841-5 (PMC9860230; doi:10.1186/s12887-023-03841-5)
Supplement: Supplementary file 4 — Additional file 4: Table S3. Adverse events occurring in ≥2% of patients in any age group (safety population). [file 12887_2023_3841_MOESM4_ESM.docx]

**Additional file 4: Table S3** Adverse events occurring in ≥2% of patients in any age group (safety population)

| **System organ class**  **Preferred term** | **Age group** | | | |
| --- | --- | --- | --- | --- |
|  | **<2 years *N* = 14** | **≥2 to <6 years *N* = 45** | **≥6 to <12 years *N* = 81** | **Overall *N* = 140** |
|  | ***n* (%)** | ***n* (%)** | ***n* (%)** | ***n* (%)** |
| Patients with any AEs^a^ | 8 (57.1) | 15 (33.3) | 32 (39.5) | 55 (39.3) |
| Infections and infestations | 5 (35.7) | 7 (15.6) | 12 (14.8) | 24 (17.1) |
| Bronchitis | 1 (7.1) | 1 (2.2) | 1 (1.2) | 3 (2.1) |
| Nasopharyngitis | 1 (7.1) | 2 (4.4) | 0 | 3 (2.1) |
| Pharyngitis | 0 | 0 | 3 (3.7) | 3 (2.1) |
| Otitis media | 1 (7.1) | 1 (2.2) | 0 | 2 (1.4) |
| Sinusitis | 0 | 0 | 2 (2.5) | 2 (1.4) |
| Upper respiratory tract infection | 2 (14.3) | 0 | 0 | 2 (1.4) |
| Bacterial infection | 0 | 0 | 2 (2.5) | 2 (1.4) |
| Oral herpes | 0 | 0 | 2 (2.5) | 2 (1.4) |
| Conjunctivitis | 0 | 1 (2.2) | 0 | 1 (0.7) |
| Influenza | 0 | 1 (2.2) | 0 | 1 (0.7) |
| Otitis media acute | 1 (7.1) | 0 | 0 | 1 (0.7) |
| Parotitis | 0 | 1 (2.2) | 0 | 1 (0.7) |
| Nervous system disorders | 0 | 0 | 2 (2.5) | 2 (1.4) |
| Headache | 0 | 0 | 2 (2.5) | 2 (1.4) |
| Respiratory, thoracic and mediastinal disorders | 1 (7.1) | 3 (6.7) | 2 (2.5) | 6 (4.3) |
| Upper respiratory tract inflammation | 0 | 2 (4.4) | 0 | 2 (1.4) |
| Asthma | 0 | 1 (2.2) | 0 | 1 (0.7) |
| Cough | 1 (7.1) | 0 | 0 | 1 (0.7) |
| Gastrointestinal disorders | 2 (14.3) | 5 (11.1) | 16 (19.8) | 23 (16.4) |
| Vomiting | 2 (14.3) | 3 (6.7) | 9 (11.1) | 14 (10.0) |
| Constipation | 0 | 1 (2.2) | 2 (2.5) | 3 (2.1) |
| Diarrhea | 0 | 0 | 3 (3.7) | 3 (2.1) |
| Dental caries | 0 | 1 (2.2) | 0 | 1 (0.7) |
| Skin and subcutaneous tissue disorders | 2 (14.3) | 2 (4.4) | 1 (1.2) | 5 (3.6) |
| Dry skin | 1 (7.1) | 1 (2.2) | 0 | 2 (1.4) |
| Eczema asteatotic | 1 (7.1) | 0 | 0 | 1 (0.7) |
| Rash | 0 | 1 (2.2) | 0 | 1 (0.7) |
| Investigations | 1 (7.1) | 0 | 2 (2.5) | 3 (2.1) |
| Platelet count increased | 1 (7.1) | 0 | 0 | 1 (0.7) |
| Injury, poisoning and procedural complications | 0 | 0 | 2 (2.5) | 2 (1.4) |
| Ligament sprain | 0 | 0 | 2 (2.5) | 2 (1.4) |

Preferred term by Medical Dictionary for Regulatory Activities Version 19.1

AE: adverse event

^a^ Includes all AEs regardless of frequency
