# Supplementary material for: Baloxavir safety and clinical and virologic outcomes in influenza virus-infected pediatric patients by age group: age-based pooled analysis of two pediatric studies conducted in Japan
Source: BMC Pediatr. 2023 Jan 21;23:35. doi: 10.1186/s12887-023-03841-5 (PMC9860230; doi:10.1186/s12887-023-03841-5)
Supplement: Supplementary file 5 — Additional file 5: Table S4. Duration of symptoms and fever by age group and influenza virus type (subtype) (ITTI population N = 137). [file 12887_2023_3841_MOESM5_ESM.docx]

**Additional file 5: Table S4** Duration of symptoms and fever by age group and influenza virus type(subtype) (ITTI population *N* = 137)

| **Influenza virus type(subtype)** | **Age group** | **Duration of symptoms**  **(TTIA)** | | **Duration of fever**  **(TTRF)** | |
| --- | --- | --- | --- | --- | --- |
|  |  | ***N*** | **Median time (hours)**  **(95% CI)** | ***N*** | **Median time (hours)**  **(95% CI)** |
| A(H1N1)pdm09 | <6 years | 9 | 58.9 (14.9–170.2) | 9 | 45.7 (14.9–59.1) |
|  | ≥6 to <12 years | 4 | 152.9 (45.3–177.1) | 4 | 33.7 (23.5–45.3) |
| A(H3N2) | <6 years | 27 | 38.9 (24.4–69.6) | 27 | 29.8 (19.8–38.2) |
|  | ≥6 to <12 years | 68 | 46.5 (33.9–62.5) | 68 | 20.6 (19.1–22.4) |
| B | <6 years | 16 | 41.8 (26.4–86.9) | 16 | 32.2 (22.5–38.2) |
|  | ≥6 to <12 years | 4 | 44.7 (18.3–81.7) | 4 | 20.6 (7.3–39.7) |

Patients who did not experience alleviation of influenza illness or resolution of fever by the last observation time point were censored at the last observation time point

CI: confidence interval; ITTI: intention-to-treat infected; TTIA: time to illness alleviation; TTRF: time to resolution of fever
