# Supplementary material for: Baloxavir safety and clinical and virologic outcomes in influenza virus-infected pediatric patients by age group: age-based pooled analysis of two pediatric studies conducted in Japan
Source: BMC Pediatr. 2023 Jan 21;23:35. doi: 10.1186/s12887-023-03841-5 (PMC9860230; doi:10.1186/s12887-023-03841-5)
Supplement: Supplementary file 6 — Additional file 6: Table S5. List of IRBs that gave ethical approval for the clinical trials JapicCTI-163,417 and JapicCTI-173,811. [file 12887_2023_3841_MOESM6_ESM.docx]

**Additional file 6: Table S5** List of IRBs that gave ethical approval for the clinical trials JapicCTI-163417 and JapicCTI-173811

| **IRBs for JapicCTI-163417** | **IRBs for JapicCTI-173811** |
| --- | --- |
| ^a^Ekihigashi Dermatology Allergy Clinic IRB | ^a^Ekihigashi Dermatology Allergy Clinic IRB |
| ^b^Fukui General Hospital IRB | ^b^Fukui General Hospital IRB Y |
| Hakodate Central General Hospital IRB | ^c^Jimbo Orthopedics IRB |
| Haradoi Hospital IRB | ^d^Medical Corporation Shintokai Yokohama Minoru Clinic IRB |
| ^e^Hayashi Diabetic Medicine Clinic IRB | Shizuoka Welfare Hospital IRB |
| KKR Sapporo Medical Center IRB | ^f^Suzuki Internal and Circulatory Medical Clinic IRB |
| Kure Medical Center IRB |  |
| Mizushima Central Hospital IRB |  |
| ^g^Nakameguro Atlas Clinic IRB |  |
| ^h^Nihonbashi Sakura Clinic IRB |  |
| Okayama City Hospital IRB |  |
| Review Board of Human Rights and Ethics for Clinical Studies IRB |  |
| Shikoku Medical Center for Children and Adults IRB |  |
| ^f^Suzuki Internal and Circulatory Medical Clinic IRB |  |
| Takatsuki General Hospital IRB |  |

IRB: Institutional Review Board

^a^This IRB oversaw ethical approval for five study sites in JapicCTI-163417 and two sites in JapicCTI-173811

^b^This IRB oversaw ethical approval for one study site in JapicCTI-163417 and two study sites in JapicCTI-173811

^c^This IRB oversaw ethical approval for nine study sites in JapicCTI-163417

^d^This IRB oversaw ethical approval for five study sites in JapicCTI-173811

^e^This IRB oversaw ethical approval for eight study sites in JapicCTI-163417

^f^This IRB oversaw ethical approval for three study sites in JapicCTI-163417 and one study site in JapicCTI-173811

^g^This IRB oversaw ethical approval for six study sites in JapicCTI-163417

^h^This IRB oversaw ethical approval for nine study sites in JapicCTI-163417
